# Environmentally relevant concentrations of thiamethoxam cause cytotoxic and genotoxic damage in bullfrog (*Lithobates catesbeianus*) tadpoles

**DOI:** 10.1007/s10646-026-03154-0

**Published:** 2026-10-01

**Authors:** Aline Arantes De Oliveira, Miller Caldas Barradas, Acácio Arlem Tomaz, Diego da Silva Lima, João Victor Borges Assis, Andréa Rodrigues Chaves, Thiago Bernardi Vieira, Marta Sofia Monteiro, Renato Almeida Sarmento, Althiéris de Souza Saraiva, Daniela De Melo e Silva

**Affiliations:** 1 Laboratory of Mutagenesis (LABMUT), Institute of Biological Sciences (ICB I), Center of Excellence in One Health (CESU), Federal University of Goiás (UFG), Goiânia, Goiás, Brazil; 2 Research group, Conservation of Agroecosystems and Ecotoxicology (CAE), Campos Belos, Goiás, Brazil; 3 Department of Biology and Center for the Study of the Environment and the Sea (CESAM), University of Aveiro (UA), Aveiro, Portugal; 4 Laboratory of Mutagenesis (LABMUT), Institute of Biological Sciences (ICB I), Federal University of Goiás (UFG), Goiás, Goiânia Brazil; 5 Center of Excellence in Molecular Studies, Energy and Petroleum (CEMEP), Institute of Chemistry (IQ), Federal University of Goiás (UFG), Goiânia, Goiás, Brazil; 6 Laboratory of Chiropteran Studies (LABEQ), Faculty of Biological Sciences (FCB), Federal University of Pará (UFPA), Altamira University Campus, Altamira, Pará, Brazil; 7 Postgraduate Program in Plant Production (PPGPV), Applied and Functional Ecology Laboratory, Federal University of Tocantins (UFT), National Institute of Science and Technology (INCT), Gurupi University Campus, Tocantins Gurupi, Brazil; 8 Federal Institute of Goiás (IF Goiano), Campos Belos Campus, research group, Conservation of Agroecosystems and Ecotoxicology (CAE), Campos Belos, Goiás, Brazil

**Keywords:** Amphibian, Anuran, DNA damage, Ecotoxicology, Neonicotinoid

## Abstract

Neonicotinoids are emerging pollutants of major concern due to their widespread use and potential risks to biodiversity, yet their genetic and lethal toxicity, key parameters in environmental risk assessment, are still poorly explored in anurans. In this study, we evaluated the acute toxicity of thiamethoxam (96 h) in *Lithobates catesbeianus* tadpoles, determining lethal concentrations (LC_10−96 h_, _50–96 h_, _90–96 h_) and assessing subindividual effects, including genotoxicity (Comet assay), mutagenicity (Micronucleus test), and cytotoxicity, at environmentally relevant concentrations (lethal: 14.40–3516 µg a.i./L; subindividual: 9.4–59 µg a.i./L). The LC_10−96 h_, _50–96 h_, _90–96 h_ were defined as 624.44, 1070.13, and 1833.03 µg a.i./L, confidence interval 95%, respectively. Subindividual exposure induced significant genotoxic effects at a concentration of 31 µg a.i./L, and mutagenic responses with an increased frequency of lobed nuclei, nuclear budding, and anucleated cells, especially at a concentration of 59 µg a.i./L. A positive correlation was observed between Comet assay parameters and nuclear abnormalities. Taken together, these findings demonstrate dose-dependent genomic damage and suggest links between distinct cellular alterations. Integrated with the lethal toxicity data, the results indicate that thiamethoxam disrupts essential cellular processes in amphibians, highlighting *L. catesbeianus* as a sensitive bioindicator, reinforcing the relevance of genotoxic and mutagenic biomarkers in assessing the impacts of neonicotinoids on aquatic ecosystems.

**Supplementary Information:**

The online version contains supplementary material available at https://doi.org/10.1007/s10646-026-03154-0.

## Introduction

The increasing use of neonicotinoid insecticides in agriculture has raised substantial concerns regarding their potential adverse effects on terrestrial and aquatic ecosystems (Zhang et al. 2018; Campbell et al. 2022; Zhou et al. 2023a). Among the organisms particularly vulnerable to exposure with these compounds are anuran amphibians (toads, frogs and tree frogs), which possess highly permeable skin and exhibit a complex life cycle that depends on both aquatic (larval) and terrestrial (adult) environments, increasing their exposure to a wide range of environmental stressors (Pombal and Haddad 2005; Zhou et al. 2023b; Shinya et al. 2023; Lopes et al. 2023; Awkerman et al. 2024). This physiological and ecological sensitivity allows them to exhibit early biological responses to pollutants, including DNA damage, oxidative stress, liver alterations, and disruptions in reproductive characteristics such as sex ratio, vocalization behavior, and offspring fitness (Zamora-Gamacho et al. 2022; Orton et al. 2023; Awkerman et al. 2024). Consequently, anurans are widely recognized as important sentinel organisms, providing early warning signals of environmental degradation (Hocking and Babbitt 2014). In recent decades, these animals have shown a sharp population decline on a global scale, a phenomenon that may be related, among other factors, to the increasing presence of emerging pollutants, such as neonicotinoids (Brühl et al. 2013; Zhu and Wang 2025).

Neonicotinoids represent a class of insecticides present in more than 120 countries (Morrissey et al. 2015; Rohonczy et al. 2020), primarily for their high efficacy (Robinson et al. 2017; Yermolenko et al. 2022), rapid absorption (Zhu and Wang 2025), chemical stability, and resistance to degradation (Yang et al. 2025). They are commonly applied to control insect pests in agricultural settings and for veterinary purposes (Wang et al. 2018; Klingelhöfer et al. 2022; Zhu and Wang 2025). Among this group, thiamethoxam is one of the most widely used neonicotinoids worldwide, with extensive application in agricultural systems (Li et al. 2025b). However, their physical and chemical properties and long half-lives eventually contribute to the persistence of these compounds in the environment, resulting in the formation of residues with the potential for environmental contamination (Simon-Delso et al. 2015; Lewis et al. 2016).

Consistent with its widespread use, thiamethoxam has been frequently detected in aquatic environments, reflecting its high environmental mobility and persistence. High detection frequencies and environmentally relevant concentrations (concentrations found in ecosystems) have been reported in surface waters and sediments across agricultural regions (in the range of 0,001 µg/L to 225 µg/L), indicating a substantial potential for aquatic exposure (Morrissey et al. 2015; Liu et al. 2021; Li et al. 2025a). Moreover, although thiamethoxam acts as a partial agonist of nicotinic acetylcholine receptors (nAChRs), its major environmental metabolite, clothianidin, exhibits stronger agonist activity, thereby increasing the ecological risks associated with neonicotinoid contamination (Nauen et al. 2003; Simon-Delso et al. 2015; Cartereau et al. 2018; Ihara and Matsuda 2018; Siviter et al. 2022; SYNGENTA 2024a).

In this context, recent studies indicate that neonicotinoids cause increased mortality at varying concentrations, in addition to potentially triggering genotoxic and cytogenetic damage in tadpoles of different species, such as *Pelophylax nigromaculatus*, *Hypsiboas pulchellus*,* Leptodactylus luctator* and *Physalaemus cuvieri* (Feng et al. 2004; Pérez-Iglesias et al. 2014; Samojeden et al. 2022). The observed effects include: (i) a dose-dependent increase in DNA damage in acute 96 h assays (Feng et al. 2004); (ii) increased frequency of micronuclei after 96 h of exposure, genetic damage detected after 48 h, and an increased frequency of DNA damage at the 96 h (Pérez-Iglesias et al. 2014); and (iii) increased occurrence of micronuclei after 7 days of exposure in *L. luctator* and in *P. cuvieri* (Samojeden et al. 2022).

Given the widespread occurrence, persistence, and documented toxicological effects of neonicotinoids in aquatic environments, the selection of sensitive and ecologically relevant bioindicator species is essential for accurately assessing their environmental risks. The amphibian *Lithobates catesbeianus*, commonly referred to as the American bullfrog, has been used as a biological indicator of environmental contamination due to its morphophysiological characteristics, susceptibility, and sensitivity to environmental changes (Motta et al. 2020; DOTS 2025; BRAZIL 2025a, 2025b). Native to North America, this species has been introduced to over 40 countries due to its economic potential (Montalvão et al. 2017). Additionally, *L. catesbeianus* has been prominent as an experimental model in ecotoxicological studies, successfully used in research involving xenobiotics such as pesticides (Rios et al. 2017; Motta et al. 2020, 2023; Carvalho et al. 2025).

In ecotoxicological assessments, the combined evaluation of survival and sub-individual damage plays a fundamental role in understanding the effects of chemical or physical agents on living organisms exposed to natural or induced environmental stressors (Magalhães and Ferrão Filho 2008). Furthermore, the gaps identified in a previous study (Oliveira et al. 2025), particularly the scarcity of research addressing the genotoxic, mutagenic, and cytogenetic effects of thiamethoxam in amphibians, reinforce the relevance and need for investigations such as the one proposed herein. In this context, the present study, using a thiamethoxam-based product, a neonicotinoid insecticide widely applied across different crops, expands current knowledge by integrating lethal toxicity thresholds with acute sub-individual biomarkers, including genotoxicity (Comet assay) and mutagenicity (Micronucleus test), as well as by analyzing the correlation between damage derived from cytogenetic biomarkers, and also cytotoxicity in *L. catesbeianus* tadpoles exposed to environmentally relevant concentrations.

Therefore, this study aims to evaluate the ecotoxicity of thiamethoxam in *L. catesbeianus* tadpoles at both individual and subindividual levels by determining acute lethal concentrations (LC_10−96 h_, _50–96 h_, _90–96 h_) and assessing genotoxic, mutagenic, and cytotoxic damage. The findings are expected to provide a scientific basis for understanding the impacts of neonicotinoids on this amphibian species and to contribute to the advancement of ecotoxicology, anuran conservation, and environmental risk assessment related to emerging contaminants in freshwater ecosystems.

## Materials and methods

### Tested insecticides

The commercial formulation based on thiamethoxam, produced by Syngenta Crop Protection Ltd., São Paulo, Brazil, contains 350 g/L (35% m/v) of 3-(2-chloro-1,3-thiazol-5-ylmethyl)-5-methyl-1,3,5-oxadiazinan-4-ylidene (thiamethoxam) and 830 g/L (83% m/v) of unspecified ingredients. A 100 mL volume of this formulation was provided by the Graduate Program in Plant Production at the Federal University of Tocantins (UFT).

To obtain the test concentrations required for this study, a stock solution was prepared at a concentration of 97.3 mg a.i./L using distilled water (Table 1). The total volume of the commercial product used to prepare the stock solution did not exceed 3 mL, which is within the available volume (100 mL of commercial product; additional details are provided in the Supporting Information).

**Table 1:** Concentrations of thiamethoxam in water samples (mg L⁻¹ and µg L⁻¹)

Samples	Concentration (mg L⁻¹, mean ± SD, *n* = 3)
A1	4.8 ± 1.4
A2	17 ± 3.0
A3	46 ± 6.8
A4	97 ± 6.6

Samples	Concentration (µg L⁻¹, mean ± SD, *n* = 3)
C1	9.4 ± 4.3
C2	31 ± 0.2
C3	59 ± 1.9

Values are expressed as mean ± Standard Deviation (SD), (*n* = 3)

Serial dilutions were then performed in naturally dechlorinated water from the 97.3 mg a.i./L stock solution to achieve the test concentrations (see Preparation calculations in the Supporting Information). During the preparation process, the physicochemical characteristics of the formulation (volatility, color, and viscosity) and the limitations of the available glassware and pipettes were carefully considered. The reference solutions were then stored at 4 °C in the dark to avoid degradation of the active ingredient.

### Chemical analysis

Chemical analyses were conducted at the Center of Excellence in Molecular Studies, Energy and Petroleum (CEMEP), Federal University of Goiás (UFG, Brazil). Certified reference material of thiamethoxam (purity ≥ 98%) was obtained from Sigma-Aldrich (St. Louis, MO, USA). Thiamethoxam concentrations in exposure water were determined by LC-MS/MS. Water samples were filtered (0.22 μm PVDF) and analyzed using an Agilent 1290 Infinity II Bio LC system coupled to an Agilent 6495D triple-quadrupole mass spectrometer. Quantification was performed using dynamic multiple reaction monitoring (dMRM), with melatonin-d₄ as the internal standard. External calibration curves were prepared in ultrapure water over the range of 5-100 ng mL⁻¹ (R² > 0.995). Samples exceeding the calibration range were appropriately diluted prior to analysis. Detailed method information, performance parameters, including chromatographic conditions, recovery, and limits of quantification, are provided in the Supporting Information (other information and Table S3).

### Acquisition and maintenance of test organisms

Organisms were acquired from a breeding facility located in Goiânia, Goiás, Brazil (16° 27′ 48′′ S, 48° 38′ 43′′ W) at stage 25 of Gosner (Gosner 1960). The tadpoles had an average weight of 55 ± 0.45 mg and an average total length of 4.75 ± 0.25 cm. After collection, they were carefully transferred to the laboratory in plastic bags (Motta et al. 2020, 2023). Before the experiments, the organisms were acclimated for 30 min in the transport plastic bags and subsequently, for 2 days in 20-liter tanks containing 10 L of naturally dechlorinated water (adapted from Organisation for Economic Co-operation and Development – OECD 2009 and Amaral et al. 2022).

After this period, they were housed in tanks with dimensions of 20 cm × 10 cm × 12 cm, containing 2 L of naturally dechlorinated water, with an additional 2 days for acclimatization (Lopes et al. 2021; Motta et al. 2023). The organisms were maintained at a constant temperature of 25 ± 2 °C and subjected to a 12 h light/dark cycle to simulate environmental conditions (Lopes et al. 2021). Throughout the experimental period, water quality was monitored to ensure optimal conditions for the development and well-being of the organisms (OECD 2009).

This study was approved by the Academic Integrity Committee (CIA) and the Ethics Committee on the Use of Animals (CEUA) of the UFG, under protocol number (08/19).

### Lethal toxicity

The experiment followed the guidelines proposed by OECD (2015, 2019a b) and was conducted using seven different concentrations of thiamethoxam, in addition to an initial measurement time - T0 (6 h) (dechlorinated water - no contaminant) and a negative control - C0 (96 h) (dechlorinated water - no contaminant). The concentrations used (14.40; 36; 90; 225; 562.5; 1406; 3516 µg a.i./L, respectively) were based on environmentally relevant concentration, highlighting the value of 225 µg/L of thiamethoxam, which corresponded to the intermediate concentration in our study (Gavel et al. 2021).

The experimental design followed this model: (1) T0 consisted of 2 replicates, each containing 6 organisms (6-hour exposure). (2) C0 consisted of 3 replicates, each containing 6 organisms (96-hour exposure). (3) Each experimental concentration was replicated 5 times, with each replicate containing 6 organisms (96-hour exposure). All organisms were housed in tanks with dimensions of 20 cm × 10 cm × 12 cm containing 2 L of naturally dechlorinated water (Lopes et al. 2021). To ensure uniformity and homogeneity, the organisms and replicates were randomized in the experimental setup. All treatments, including naturally dechlorinated water with their respective test concentrations, were prepared by serial pipetting of the stock solution (using an automatic micropipette for greater precision) directly into the tanks.

During the exposure period, an alternating light (12 h) and dark (12 h) cycle was established (Lopes et al. 2021). To maintain water quality, a semi-static system was used with 70% medium renewal every 24 h. To determine the LC_10_, LC_50_ and LC_90_, data were recorded after a period of 96 h (Zhou et al. 2023a). Mortality was assessed by observing immobility.

### Sub-individual toxicity

Organisms were exposed to three different concentrations (corresponding to 1.25%, 2.5% and 5% of the LC₅₀), together with a C0, over 96 h, in addition to an initial sampling point at T0 (6 h) (methodology adapted from Pérez-Iglesias et al. 2014).

The experimental design followed the model: (1) T0 consisted of 1 replicate containing 6 organisms (6-hour exposure). (2) C0 consisted of 2 replicates, each containing 6 organisms (96-hour exposure). (3) Each experimental concentration was replicated 3 times, with each replicate containing 6 organisms (96-hour exposure). All organisms were housed in tanks measuring 20 cm × 10 cm × 12 cm containing 2 L of naturally dechlorinated water (Lopes et al. 2021). To ensure uniformity and homogeneity, organisms and replicates were randomized in the experimental arrangement. All treatments, naturally dechlorinated water together with their respective test concentrations, were prepared by serial pipetting of the stock solution, directly into the tanks. To maintain water quality, a semi-static system was used with 70% medium renewal every 24 h. After 96 h of exposure, the organisms were anesthetized (at low temperature, in Petri dishes, under crushed ice) and euthanized (under the same low temperature conditions and also by cardiac puncture), in accordance with the recommendations of the CIA and the CEUA of UFG.

### Genotoxicity assay

**Fig. 1:**
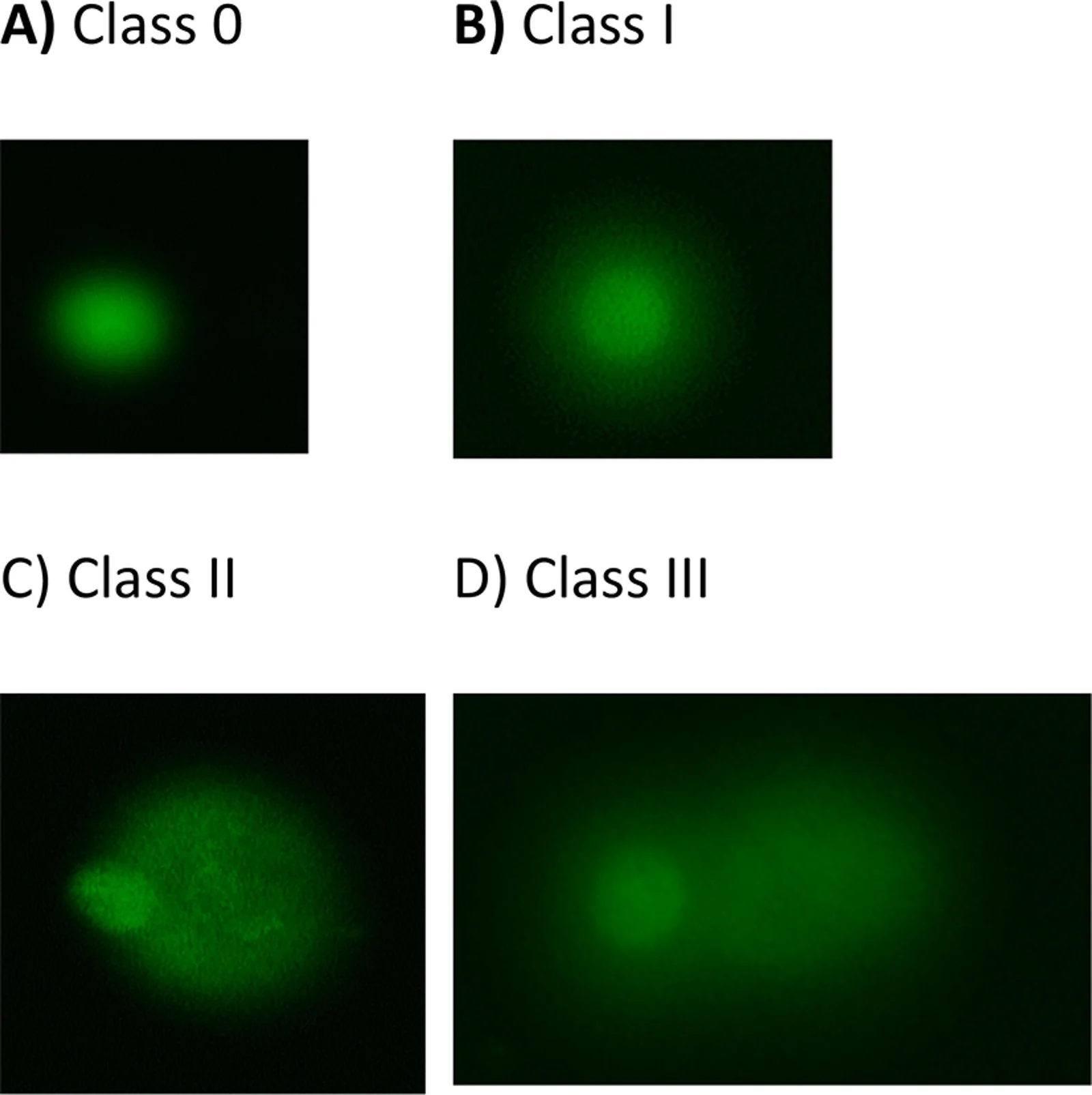
Comet assay of *Lithobates catesbeianus* after 96 h of exposure to different concentrations of the insecticide thiamethoxam (Cruiser^®^ 350 FS). Control (T0–6 h and C0–96 h) and treatment groups exposed to concentrations of thiamethoxam (µg a.i./L). (**A**) Normal cell, (**B**) Low damage, (**C**) Moderate damage, (**D**) High damage. Scored via Comet Imager software and based on: Møller et al. (41). (**A**) Class 0 (**B**) Class I (**C**) Class II (**D**) Class III

The Comet assay was performed using the method described by Singh et al. (1988) with some adjustments described by Motta et al. (2020) and scored based on Møller et al. (2020) and scores based on Møller et al. (2023) (Fig. 1). A whole blood sample (12 µL) was collected from the base of the tail of each tadpole (at the time of euthanasia) and resuspended in 1.5 mL of phosphate-buffered saline (PBS). An aliquot of the cell suspension was mixed with an equal volume (12 µL) of low-melting-point agarose (0.1%, maintained at 40 °C) and deposited onto glass slides coated with 1.5% normal-melting-point agarose. The slides were solidified at 4 °C (5 min.), lysed in ice-cold buffer (2.5 M NaCl, 100 mM EDTA, 10 mM Tris-HCl, 1% Triton X-100, 10% DMSO; pH 10) for 15 h (6 °C) in the dark, and subsequently subjected to alkaline unwinding (300 mM NaOH and 200 mM EDTA, pH > 13) and electrophoresis (25 V, 300 mA, 30 min). After electrophoresis, the slides were neutralized, fixed in absolute ethanol, air-dried, and stained with SYBR^®^ Gold I.

Comets were visualized using an Imager D2 epifluorescence microscope (Carl Zeiss, Germany) and analyzed with Comet Imager software (version 2.2; Meta Systems GmbH). For each experimental group, eight animals were evaluated, with 200 nuclei analyzed per animal (two slides), totaling 1,600 nuclei per group. All analyses were performed by a single observer. The parameters that were evaluated in the Comet assay included Tail Length, percentage of DNA in the Tail (% DNA) and the Olive Tail Moment.

### Mutagenicity assay

The mutagenicity assay (micronucleus, binucleated, nuclear bud, anucleated, apoptotic nuclei, pyknosis, notched nuclei, reniform nuclei and lobed nuclei) was performed following a protocol adapted from Borges et al. (2019), Lopes et al. (2021) and Benvindo-Souza (2020). Blood samples were collected from the tail of each tadpole by caudal prick. For each organism, two to three slides were prepared using a zigzag smear. The smears were fixed in absolute methanol for 10 min and air-dried at room temperature (27 °C). Subsequently, the slides were stained with a panoptic kit by sequential immersion in dyes 1, 2, and 3 for 5, 10, and 15 s, respectively. After staining, the slides were again air-dried at room temperature (27 °C). A total of 2000 erythrocytes (per organism) were examined under a Zeiss optical microscope at 100× magnification.

### Statistical data analysis

Probit analysis was used to estimate the LC10-96 h, LC50-96 h, and LC90-96 h values (Zhou et al. 2023b), using the statistical software Minitab 18 (Minitab Inc., State College, PA, USA).

All cytogenetic analyses (of DNA damage biomarkers) were performed in the R statistical environment (R Core Team 2025). For the analyses between treatment groups in the genotoxic tests (all parameters of the Comet assay) and mutagenic tests (all analyses of the Micronucleus test), we used Permutational Multivariate Analysis of Variance (PERMANOVA) with distance matrices (Anderson 2001). This analysis aims to assess whether the group means are significantly different, allowing us to determine if the genotoxic and mutagenic effects present distinct mean values between the controls (T0 and C0) and the thiamethoxam treatments. We performed PERMANOVA using Euclidean distance and presented the results using Principal Coordinate Analysis (PCoA) plots (Anderson 2006).

PERMANOVA was performed using the adonis2 function, permutational dispersion analysis (Permdisp) with the betadisper function. The Euclidean distance matrix was generated with the vegdist function, implemented in the vegan package (Oksanen et al. 2025). To minimize the impact of extreme values, the data were previously logarithmically transformed (natural transformation - ln[x + 1]), and the analyses were conducted separately for genotoxic and mutagenic data derived from Comet and Micronucleus tests. After identifying the general pattern, we applied PERMANOVA to each variable, allowing us to evaluate the effect of the treatments on each of them. The effect sizes were represented in box plots, and pairwise comparison tests were conducted between each combination of treatments. To explore the correlation (of the biomarkers evaluated) between genotoxic and mutagenic damage, we performed a Two-Configuration Procrustes Rotation (Peres-Neto and Jackson 2001), comparing the rankings of genotoxic variables with mutagenic variables. We considered only the significant variables identified in the previous step and used the first two axes of the PCoA for each variable. The correlation between genotoxic (Comet assay) and mutagenic (Micronucleus test) effects was measured using the protest function, also from the vegan package.

The physicochemical parameters of the water (pH, dissolved oxygen, conductivity, turbidity, temperature, and ammonia) were analyzed using GraphPad Prism version 10 (GraphPad Software, La Jolla, California, USA). The data were examined to identify outliers and assessed for normality and homogeneity of variances. As the parametric assumptions were not met, non-parametric statistical approaches were adopted. Differences between treatments were tested using the Kruskal-Wallis test, followed by Dunn’s post-hoc test in relation to group C0. The temporal variation of the physicochemical parameters during the exposure period (0–96 h) was evaluated using non-parametric ANOVA - Friedman test, followed by Dunn’s post-hoc test for multiple comparisons with group C0.

All box plots (of biomarkers and physicochemical parameters) were generated using GraphPad Prism software to facilitate data visualization and improve graphical interpretation.

## Results

**Fig. 2:**
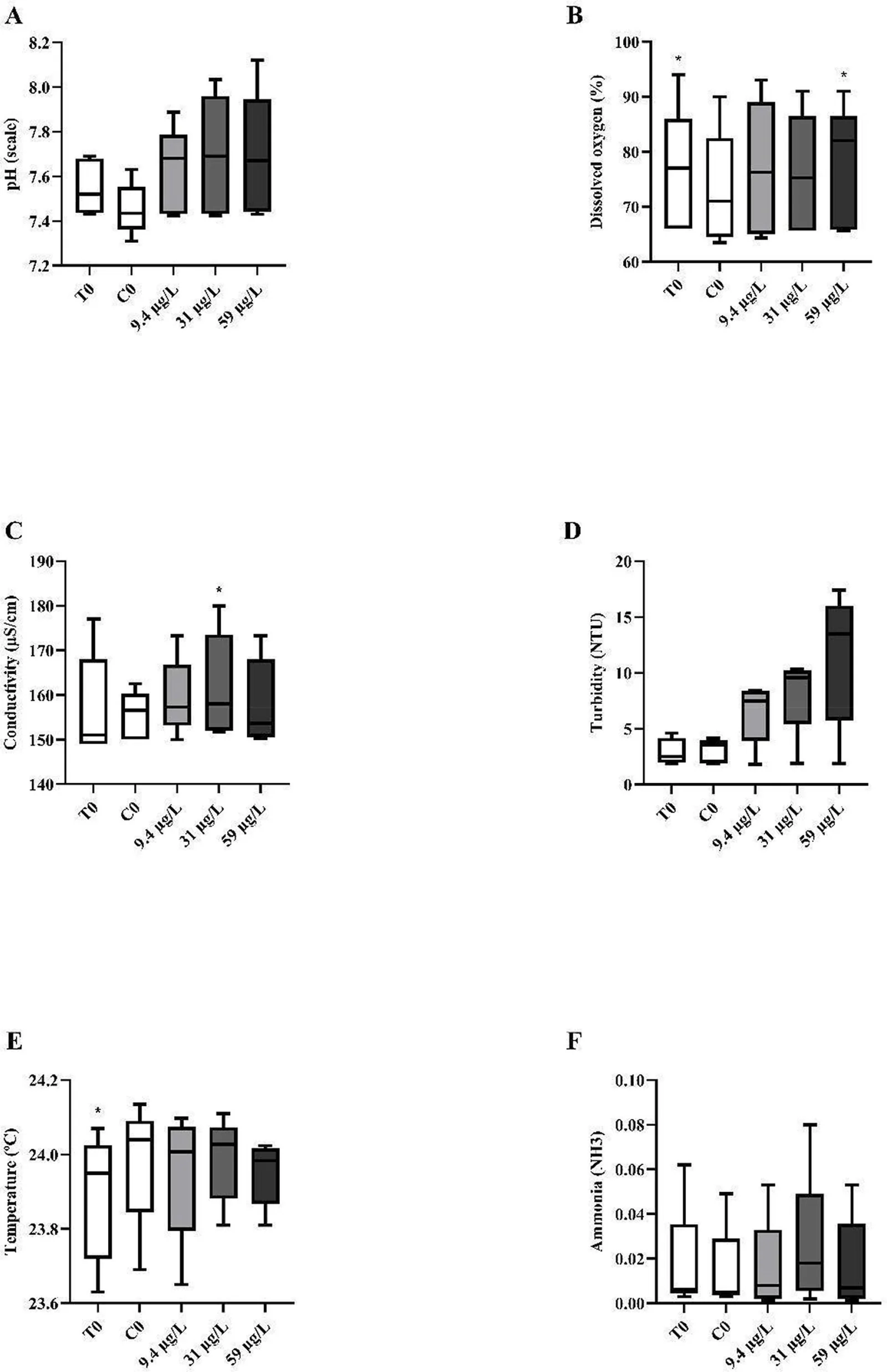
Boxplot representation of general trends in water physicochemical parameters during the experimental period (0–96 h) under exposure to different concentrations of the insecticide thiamethoxam (Cruiser^®^ 350 FS). Control (T0 and C0) and treatment groups exposed to concentrations of thiamethoxam (µg a.i./L). Parameters analyzed: (**A**) pH, (**B**) Dissolved oxygen (mg/L), (**C**) Conductivity (µS/cm), (**D**) Turbidity (NTU), (**E**) Temperature (°C), and (**F**) Ammonia (NH3). *Indicates significant differences compared to the C0 (*p* < 0.05; nonparametric ANOVA - *Friedman tests*, followed by *Dunn’s* post-hoc test)

The evaluation of the physicochemical parameters of the water during the exposure period (0–96 h) in the subindividual test with thiamethoxam is presented in Fig. 2. In general, the parameters remained stable between treatments, presenting discrete variations. Dissolved oxygen showed a significant increase at the highest concentration tested (59 µg a.i./L) compared to the C0. The increase in dissolved oxygen observed at the highest concentration of thiamethoxam may be associated with reduced oxygen consumption by organisms exposed under toxicological stress, reflecting possible alterations in respiratory and metabolic activity (Paunescu et al. 2022). Conductivity increased at the intermediate concentration (31 µg a.i./L), while temperature differences were observed only in the T0-6 h without compromising the overall interpretation of the results. No significant differences were detected for pH and ammonia.

In addition, detailed analyses of individual physicochemical parameters are provided in Tables S4-S9 (Supporting Information). Turbidity showed a significant increase at 59 µg a.i./L after 24 h (17.43 ± 0.42 vs. 1.85 ± 0.07 in C0) and 72 h (14.63 ± 0.91 vs. 3.75 ± 0.21 in C0). Dissolved oxygen increased significantly at 9.4 µg a.i./L after 48 h (85 ± 1.00 vs. 75 ± 0.00 in C0) and after 96 h (93 ± 0.01 vs. 90 ± 0.01 in C0). pH showed a significant increase at only two time points: 59 µg a.i./L after 72 h (8.12 ± 0.03 vs. 7.47 ± 0.04) and 31 µg a.i./L after 96 h (7.69 ± 0.01 vs. 7.63 ± 0). Conductivity, temperature, and ammonia showed no significant changes throughout the experimental period. Despite the observed differences, the physicochemical parameters remained within ranges compatible with aquatic ecotoxicological assays, including dissolved oxygen saturation above 60% and stable pH conditions throughout the experimental period, in accordance with international recommendations for acute aquatic toxicity testing (OECD 2019a). Overall, the observed physicochemical fluctuations were transient and did not compromise water quality, as reflected by the mean values and standard deviations reported in tables (S4-S9 in Supporting Information). This pattern is consistent with the semi-static system adopted, with a 70% renewal of the medium every 24 h, which contributed to the rapid stabilization of the physicochemical conditions throughout the experimental period.

In the chemical analysis, thiamethoxam was quantified in all analyzed water samples, with concentrations spanning two orders of magnitude, from as low as µg L⁻¹ to mg L⁻¹ (Table 1). The method exhibited excellent analytical performance, as summarized in the Supporting Information (Table S10). Calibration was linear within the range of 5-100 ng mL⁻¹ (R² = 0.9989), with a estimated LOQ of 0.39 ng mL⁻¹, ensuring adequate sensitivity for monitoring thiamethoxam in environmental water samples. Recoveries were concentration-dependent but remained within acceptable ranges, from 72% at the lowest fortification level (5 ng mL⁻¹) to nearly quantitative values at higher concentrations (90% and 99% for 25 and 100 ng mL⁻¹, respectively). These results confirm the accuracy of the method for both trace- and high-level determinations.

Representative TICs of ultrapure water (blank), spiked water, and a real sample are shown in the Supporting Information (Figure S1). The absence of interfering signals in the blank highlights the high selectivity of the LC-MS/MS method. Furthermore, real water samples exhibited chromatographic behavior comparable to spiked samples, confirming the unequivocal identification of thiamethoxam. Importantly, none of the additional monitored pesticides were detected (Table S3 in the Supporting Information), reinforcing that thiamethoxam was the sole target analyte present in the analyzed samples.

The LC values for 10%, 50%, and 90% of L. catesbeianus after 96 h of exposure to thiamethoxam were estimated as 624.44 µg a.i./L (95% CI: 436.93–765.80), 1070.13 µg a.i./L (95% CI: 896.75-1275.52), and 1833.03 µg a.i./L (95% CI: 1497.53-2611.05), respectively (Table 2). During the experiment, mortality in the control group remained below 10%.

**Table 2:** Lethal concentration (LC_10− 96 h_, _50–96 h, 90–96 h; µg a.i./L_) of thiamethoxam insecticide (Cruiser® 350 FS) in *Lithobates catesbeianus* after 96 h of exposure, estimated by Probit analysis (95% confidence interval; n = 30)

LC (%)	EC (µg a.i./L)	SE (µg a.i./L)	IL (µg a.i./L)	UL (µg a.i./L)
10	624.44	80.48	436.93	765.80
50	1070.13	90.01	896.75	1275.52
90	1833.03	234.05	1497.53	2611.05

*EC* Estimated Concentration, *SD* Standard Error, *IL* Inferior limit, *UL* Upper limit

**Fig. 3:**
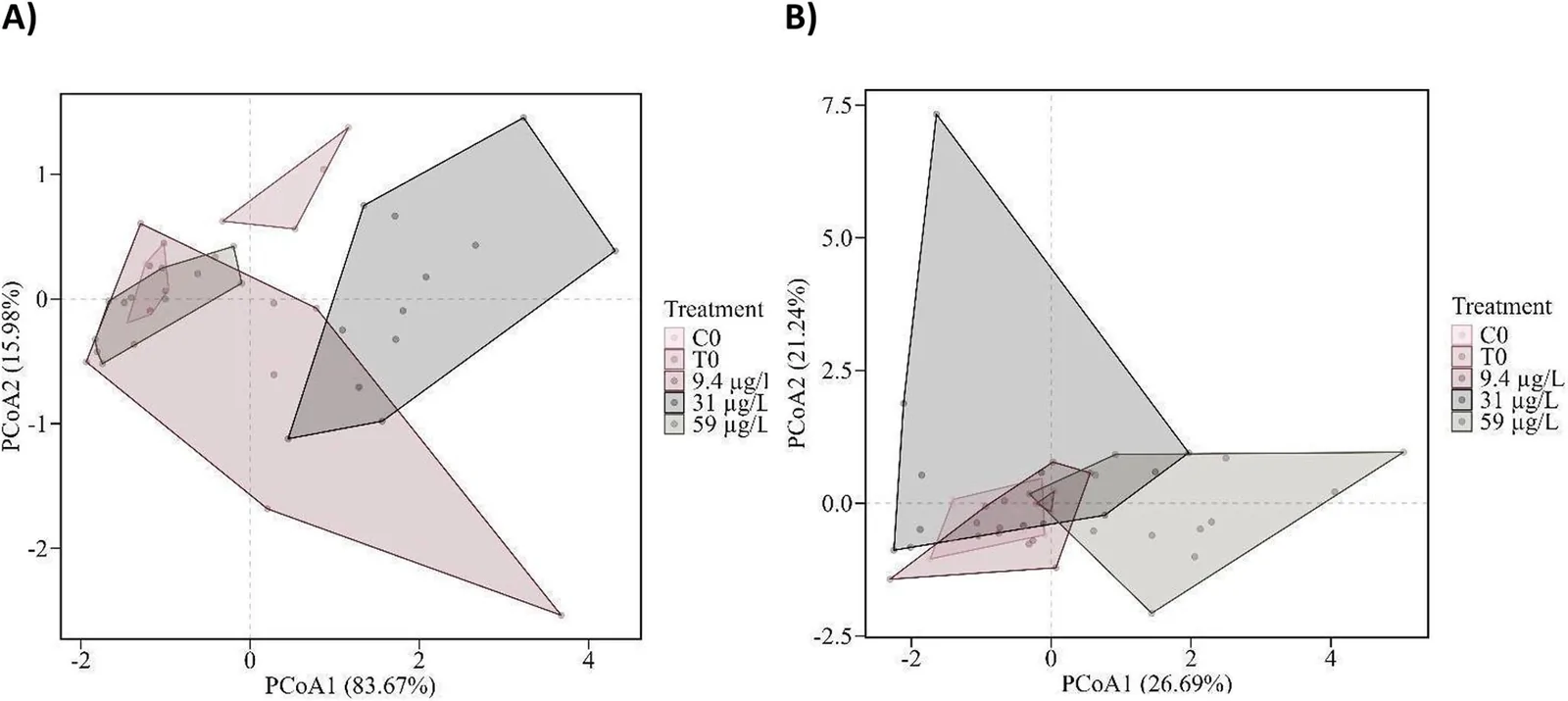
Principal Coordinates Analysis (PCoA) of (**A**) genotoxic damage (Tail Length, Olive Tail Moment, % DNA) and (**B**) mutagenicity (micronucleated, pyknosis, binucleated, nuclear bud, anucleated, apoptotic nuclei, notched nuclei, reniform nuclei, lobed nuclei) of *Lithobates catesbeianus* after 96 h of exposure to different concentrations of the thiamethoxam insecticide (Cruiser^®^ 350 FS). Control (T0 and C0) and treatment groups exposed to concentrations of thiamethoxam (µg a.i./L), (*n* = 3 for 3.4–59 µg a.i./L, *n* = 1 for T0, and *n* = 2 for C0; 4 organisms per replicate)

We observed a significant genotoxic effect in the Comet assay (F (4,46) = 13.164, R² = 0.556, p < 0.001) (Fig. 3A) and a mutagenic effect in the Micronucleus test (F (4,46) = 2.530, R² = 0.194, p < 0.001) (Fig. 3B). Overall, we found that treatments T0 and 31 µg a.i./L differed from the other treatments, while C0, 9.4 µg a.i./L, and 59 µg a.i./L exhibited a similar pattern for genotoxic effects (Fig. 3A). Regarding mutagenic effects (Fig. 3B), treatments 31 µg a.i./L and 59 µg a.i./L formed distinct groups, while the remaining groups showed a similar mutagenic response.

**Fig. 4:**
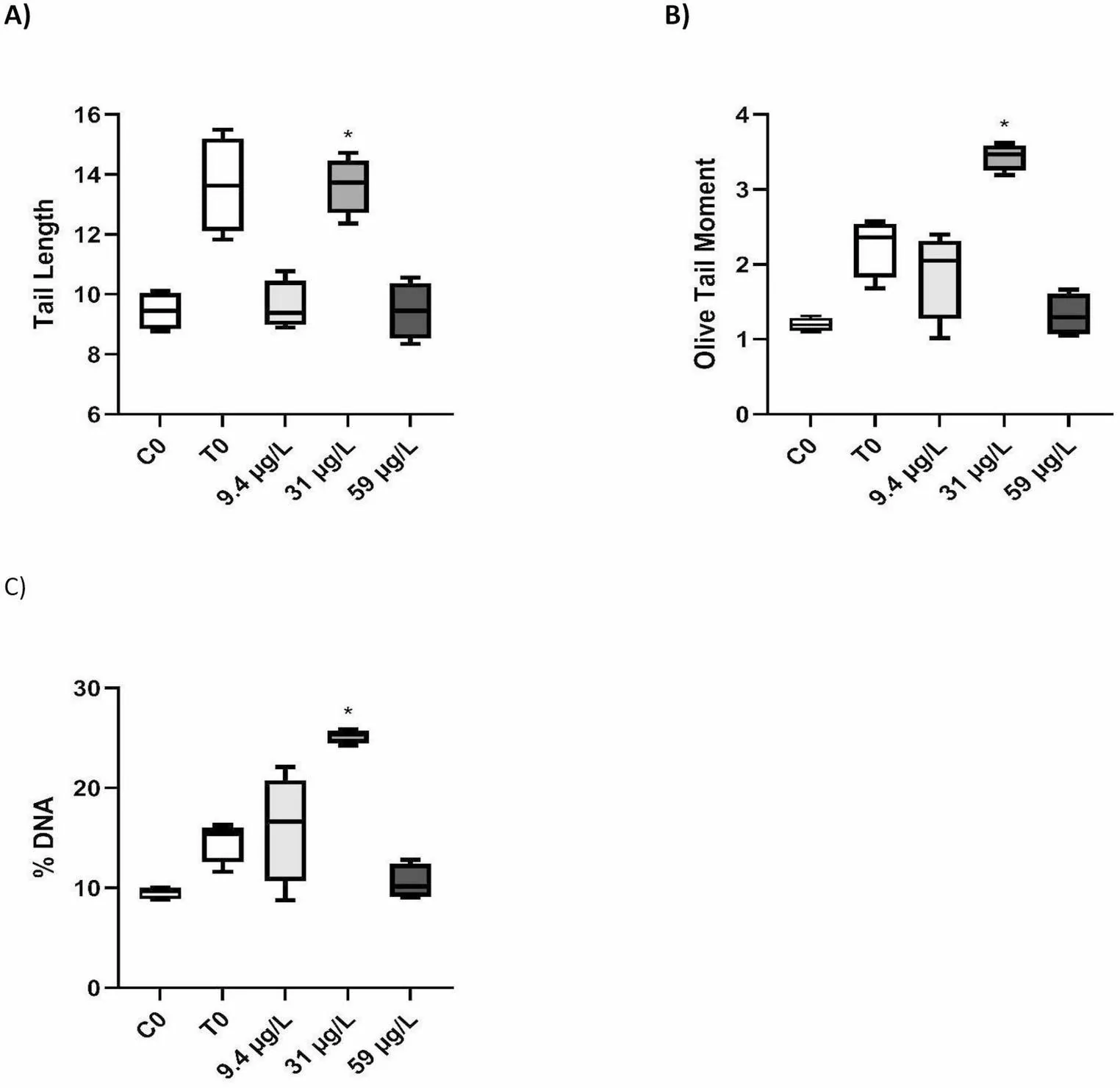
Boxplot of Comet assay for *Lithobates catesbeianus* after 96 h of exposure to different concentrations of the insecticide thiamethoxam (Cruiser^®^ 350 FS), for (**A**) Tail Length, (**B**) Olive Tail Moment, (**C**) % DNA. Control (T0 and C0) and treatment groups exposed to concentrations of thiamethoxam (µg a.i./L), (*n* = 3 for 3.4–59 µg a.i./L, *n* = 1 for T0, and *n* = 2 for C0; 4 organisms per replicate). *Indicates significant differences compared to the control (*p* ≤ 0.05; PERMANOVA)

 Regarding the individual effects tested for each variable, we observed that all genotoxic effect measures (specifically from the Comet assay) showed significant differences between treatments (Fig. 4A, B, C; Table 3). Higher values were recorded for T0 and 31 µg a.i./L, and lower values for C0, 9.4 µg a.i./L and 59 µg a.i./L, considering Tail Length (F (4,46) = 14.026, R² = 0.572, p < 0.001), Olive Tail Moment (F (4,46) = 15.257, R² = 0.592, p < 0.001), and % DNA (F (4,46) = 10.698, R² = 0.505, p < 0.001).

**Table 3:** R² values (lower triangle, below the main diagonal) and p-values (upper triangle, above the main diagonal) from pairwise tests conducted on genotoxic and cytotoxic variables with significant differences between treatments

	Grupo	9.4 µg a.i./L	31 µg a.i./L	59 µg a.i./L	T0	C0
Tail Length	9.4 µg a.i./L		0.001	0.782	0.003	0.733
	31 µg a.i./L	0.491		0.001	0.996	0.004
	59 µg a.i./L	0.003	0.509		0.002	0.895
	T0	0.608	0.000	0.622		0.005
	C0	0.008	0.500	0.001	0.816	
Olive Tail Moment	9.4 µg a.i./L		0.004	0.110	0.545	0.180
	31 µg a.i./L	0.399		0.001	0.011	0.001
	59 µg a.i./L	0.099	0.770		0.004	0.447
	T0	0.024	0.379	0.566		0.005
	C0	0.113	0.769	0.036	0.839	
% DNA	9.4 µg a.i./L		0.009	0.098	0.847	0.158
	31 µg a.i./L	0.244		0.001	0.003	0.001
	59 µg a.i./L	0.110	0.861		0.003	0.342
	T0	0.004	0.670	0.420		0.007
	C0	0.114	0.872	0.055	0.766	
Nuclear bud	9.4 µg a.i./L		0.325	0.036	0.483	0.371
	31 µg a.i./L	0.047		0.150	0.211	0.088
	59 µg a.i./L	0.195	0.102		0.138	0.024
	T0	0.056	0.138	0.180		0.838
	C0	0.051	0.169	0.265	0.008	
Anucleated	9.4 µg a.i./L		0.104	0.010	0.050	1.000
	31 µg a.i./L	0.123		0.533	1.000	0.186
	59 µg a.i./L	0.283	0.026		0.762	0.029
	T0	0.272	0.001	0.013		0.081
	C0	0.001	0.105	0.257	0.413	
Lobed nuclei	9.4 µg a.i./L		0.014	0.010	0.349	0.621
	31 µg a.i./L	0.244		0.001	0.522	0.022
	59 µg a.i./L	0.252	0.478		0.068	0.010
	T0	0.073	0.053	0.240		0.415
	C0	0.014	0.244	0.285	0.090	

Negative control (T0–6 h and C0–96 h) and experimental groups of tadpoles exposed to increasing concentrations of thiamethoxam (µg a.i./L) (Cruiser® 350 FS). The values correspond to the PERMANOVA test results

**Fig. 5:**
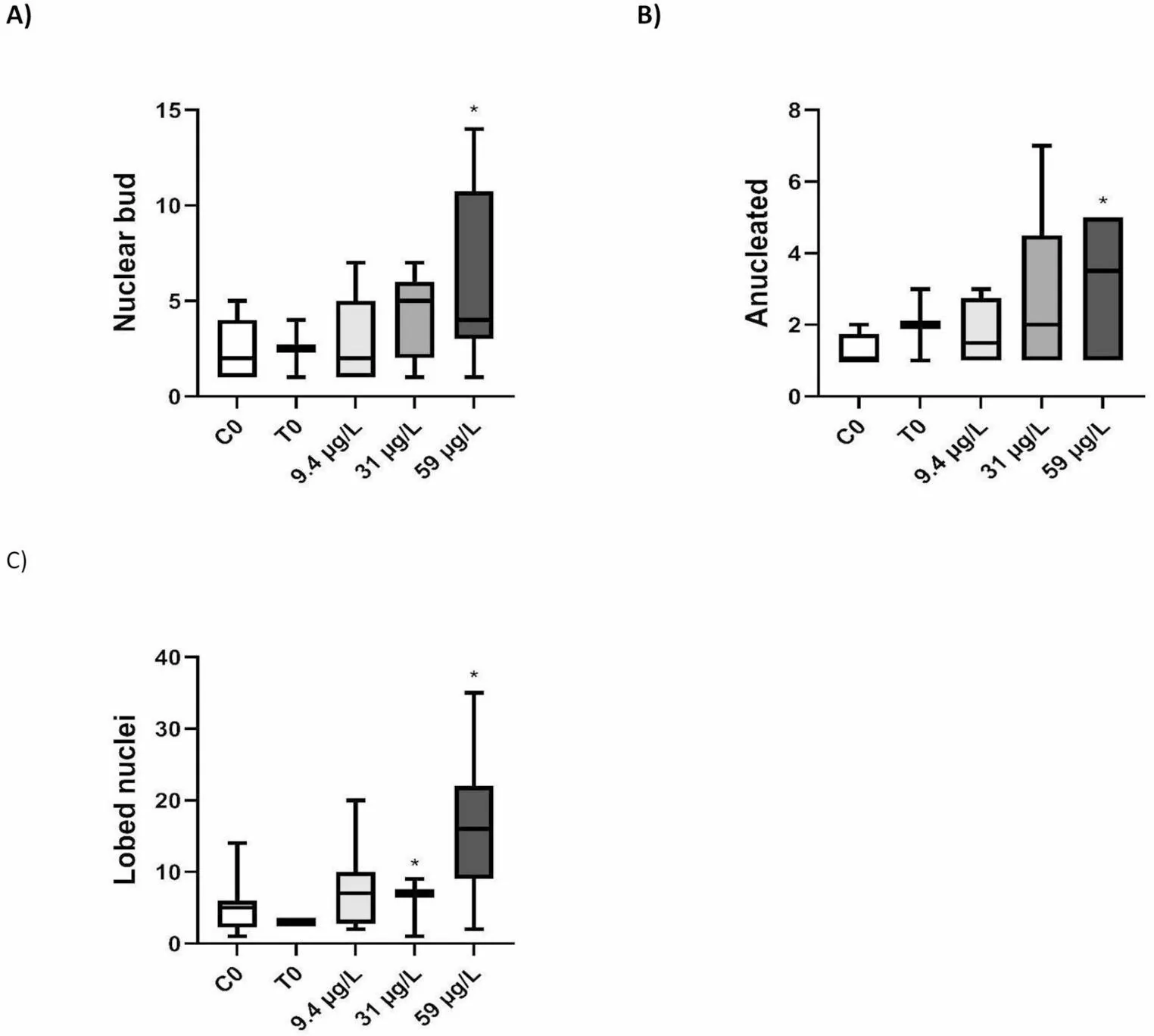
Boxplot of Micronucleus for *Lithobates catesbeianus* after 96 h of exposure to different concentrations of the insecticide thiamethoxam (Cruiser^®^ 350 FS), for (**A**) Nuclear bud, (**B**) Anucleated, and (**C**) Lobed nuclei. Control (T0 and C0) and treatment groups exposed to concentrations of thiamethoxam (µg a.i./L), (*n* = 3 for 3.4–59 µg a.i./L, *n* = 1 for T0, and *n* = 2 for C0; 4 organisms per replicate). *Indicates significant differences compared to the control (*p* ≤ 0.05; PERMANOVA)

 For mutagenic damage, significant effects were observed in nuclear buds (F (4,46) = 1.954, R² = 0.246, p = 0.023), anucleated cells (F (4,46) = 2.707, R² = 0.205, p = 0.045), and lobed nuclei (F (4,46) = 8.045, R² = 0.434, p = 0.002), with a consistent pattern across these three variables, showing the highest values in 59 µg a.i./L (Fig. 5A, B, and C; Table 3).

Additionally, in a complementary analysis, in Table 4, the 31 µg a.i./L treatment (31 µg a.i./L) presented the highest values of % DNA, Olive Tail Moment and Tail Length, confirming the genotoxic pattern observed in Figures 3A and 4A, B and C, where the 31 µg a.i./L treatment stood out in relation to the others.

**Table 4:** Genotoxic parameters of *Lithobates catesbeianus* after 96 h of exposure to insecticide thiamethoxam (Cruiser^®^ 350 FS)

Exposure Time (96 h)	T0	C0	9.4 µg a.i./L	31 µg a.i./L	59 µg a.i./L
% DNA	14.678 ± 2.085	9.525 ± 0.585	16.040 ± 5.489	25.186 ± 0.695	10.552 ± 1.804
OTM	2.244 ± 0.344	1.199 ± 0.079	1.881 ± 0.518	3.439 ± 0.157	1.326 ± 0.249
TL	13.645 ± 1.608	9.446 ± 0.638	9.607 ± 0.821	13.638 ± 0.964	9.449 ± 0.958

Negative control (T0–6 h and C0–96 h) and experimental groups of tadpoles exposed to increasing concentrations of thiamethoxam (µg a.i./L). Values are expressed as mean ± Standard Deviation (SD). *Indicates significant differences compared to the control (p ≤ 0.05; PERMANOVA)

*>OTM* Olive Tail Moment, *TL* Tail Length

Furthermore, Table 5 also presents the incidence of micronuclei and other nuclear anomalies in *L. catesbeianus*, highlighting the mutagenic effects of thiamethoxam treatments after 96 h of exposure.

**Table 5:** Incidence of micronuclei and other nuclear anomalies of *Lithobates catesbeianus* after 96 h of exposure to insecticide thiamethoxam (Cruiser^®^ 350 FS)

Exposure Time (96 h)	T0	C0	9.4 µg a.i./L	31 µg a.i./L	59 µg a.i./L
MN	0.00	0.00	0.17 ± 0.14	0.00	0.92 ± 0.63
PK	0.00	0.00	0.00	0.08 ± 0.14	0.00
BI	1.00	2.63 ± 0.53	3.83 ± 0.63	4.25 ± 1.98	2.25 ± 0.43
NB	1.25	2.00 ± 0.71	2.92 ± 2.24	3.92 ± 0.95	6.25 ± 3.36
AN	1.50	0.63 ± 0.18	0.58 ± 0.58	1.83 ± 1.25	2.50 ± 0.50
AP	17.75	17.88 ± 3.71	14.50 ± 2.05	18.33 ± 2.79	22.58 ± 3.89
ND	2.25	1.38 ± 0.18	2.33 ± 0.29	1.50 ± 1.32	3.17 ± 0.80
RN	4.50	3.50 ± 1.06	4.57 ± 1.78	*4.83 ± 1.77*	8.00 ± 3.44
LB	2.25	5.25 ± 1.06	6.42 ± 4.62	1.42 ± 1.28	16.08 ± 6.53

Negative control (T0–6 h and C0–96 h) and experimental groups of tadpoles exposed to increasing concentrations of thiamethoxam (µg a.i./L). Values are expressed as mean ± Standard Deviation (SD)

*MN* Micronucleated, *PK* Pyknosis, *BI* Binucleated, *NB* Nuclear bud, *AN* Anucleated, *AP* Apoptotic nuclei, *ND* Notched nuclei, *RN* Reniform nuclei, *LB* Lobed nuclei

Regarding the correlation between genotoxic and mutagenic variables, only the variables Tail Length (m² = 0.872, *r* = 0.356, *p* = 0.016) and Olive Tail Moment (m² = 0.908, *r* = 0.303, *p* = 0.040) showed a positive correlation with Lobed nuclei.

## Discussion

In addition to acute lethal effects at 1070.13 µg a.i./L (LC50−96 h), acute subindividual exposures of thiamethoxam induced genotoxic and mutagenic alterations in L. catesbeianus tadpoles, with distinct patterns between biomarkers (Comet vs. Micronucleus), as also observed in other neonicotinoid studies (Feng et al. 2004; Arcaute et al. 2014).

Genotoxicity detected by the Comet assay (% DNA, Olive Tail Moment, and Tail Length) peaked at 31 µg a.i./L, while mutagenic effects were most pronounced at 59 µg a.i./L, evidenced by the increased frequency of budding, anucleated and lobed nuclei. This pattern suggests mechanistic and/or kinetic differences between genotoxic and mutagenic outcomes. Furthermore, a positive correlation was observed between the DNA damage parameters of the Comet assay (Olive Tail Moment and Tail Length) and the frequency of lobed nuclei. Taken together, the results obtained at the individual and subindividual levels fill an important gap in the scientific literature (Pérez-Iglesias et al. 2014; Morais et al. 2017; Rios et al. 2017; Gavel et al. 2019; Robinson et al. 2019; Campbell et al. 2022; Mamy et al. 2025; Oliveira et al. 2025), by demonstrating the lethal, genotoxic, mutagenic, and cytotoxic effects of thiamethoxam on *L. catesbeianus*.

As widely recognized in the literature, there is considerable variability in tolerance among different species towards pesticides (Pérez-Iglesias et al. 2014; Oliveira et al. 2025). The estimated lethal concentration values for 50% of the organisms tested in this study were considerably lower than those reported for other amphibian species exposed to the same neonicotinoid. Peña et al. (2022), when evaluating South American amphibians exposed to thiamethoxam, reported LC₅₀-96 h values ranging from 11.28 to > 71.2 mg/L, depending on the species and developmental stage, values substantially higher than those observed in this study. Similar results were also recorded in studies with another neonicotinoid, imidacloprid, including *Rana limnocharis* (82 mg/L) (Feng et al. 2004), *Xenopus laevis* (74.18 mg/L) and *Rana nigromaculata* (173.55 mg/L) (Zhou et al. 2023b).

Furthermore, the estimated 96 h LC₅₀ for L. catesbeianus in the present study (1.07 mg/L) was approximately 98% lower than the LC₅₀ of 52.62 mg/L observed for *Hypsiboas pulchellus* exposed to a commercial imidacloprid formulation (Pérez-Iglesias et al. 2014), reinforcing the high acute toxicity of thiamethoxam to *L. catesbeianus*. Complementarily, Pochini and Hoverman (2017) demonstrated that prior ranavirus infection reduced the LC₅₀ of thiamethoxam in *Lithobates sylvaticus* to values close to the environmental concentrations detected in natural systems (~ 1 mg/L), suggesting that the toxicity of this insecticide may be potentiated in scenarios involving multiple stress factors.

Our tests were conducted based on the concentration of the active ingredient (thiamethoxam - Cruiser^®^ 350 FS), an approach widely adopted in ecotoxicological studies because it more realistically represents products applied in production systems and that can actually be found in the natural environment (Pérez-Iglesias et al. 2014; Morais et al. 2017; Melo et al. 2022; Samojeden et al. 2022). Nevertheless, it is important to recognize that commercial formulations contain “other ingredients,” generally consisting of components classified as inert or adjuvants, such as solvents, dispersants, surfactants, and stabilizers, which, although not intended to exert direct pesticidal activity, play an essential role in the performance, stability, and applicability of the product (OECD 2019b; SYNGENTA 2023, 2024a; BASF 2025). From a regulatory standpoint, these substances are considered inert; however, this does not imply an absence of toxicological risk, since they can influence the bioavailability, absorption, and toxicity of the active ingredient (OECD 2019b; BRAZIL 2026). Thus, the lack of transparency, resulting from regulatory guidelines, regarding the identity and proportion of these co-formulants constitutes an important limitation in studies such as this one.

However, as mentioned earlier, we observed a non-linear pattern for genotoxic effects in the 31 µg a.i./L range, where the highest levels of damage were recorded, in contrast to the response at the highest concentration tested (59 µg a.i./L). The nonlinear response observed in our results may be associated with toxicodynamic and toxicokinetic phenomena typical of neonicotinoids. Nonmonotonic dose-response relationships, often described as hormetic-like patterns, have been reported for neuroactive compounds, in which intermediate concentrations may elicit greater biological stress or instability than higher exposures, due to toxicodynamic and toxicokinetic mechanisms (Calabrese and Mattson 2017). It should also be considered that, in addition to the possible saturation of target receptors at certain concentrations of neonicotinoids (Tomizawa and Casida 2005; Gibbons et al. 2015; Lee-Jenkins and Robinson 2018), higher doses can induce such high levels of damage that they culminate in cell death (Samojeden et al. 2022). This process would reduce the proportion of viable cells, limiting the detection of genotoxic damage in bioassays and, consequently, resulting in an apparent decrease in the response at higher concentrations. However, in the present study, apoptotic nuclei did not show a statistically significant increase in the 59 µg a.i./L range. Even so, the significant increase in the frequency of anucleated nuclei may indicate the occurrence of severe cellular damage and loss of nuclear integrity, consistent with advanced cytotoxic processes. This interpretation is consistent with the recommendations of the OECD guidelines for genotoxicity assays (TG 489 - Comet assay), which warn of the risk of underestimating the response under conditions of high cytotoxicity (OECD^ 2014^).

Mechanistically, neonicotinoids are nAChR agonists designed for greater selectivity for insect targets and therefore with lower affinity for vertebrate nAChRs (Pérez-Iglesias et al. 2014; Robinson et al. 2021). However, this selectivity does not prevent toxic effects in non-target organisms. The thiamethoxam (Cruiser 350 FS) package insert describes rapid oral absorption, preferential distribution to the liver and blood, first-order clearance (half-lives of 2–6 h), and predominantly urinary excretion, with low tissue persistence after seven days, with partial biotransformation to metabolites such as clothianidin, which may exhibit greater biological activity and contribute to overall toxicity (SYNGENTA 2024b). Although thiamethoxam exhibits lower agonist activity on nAChRs in vertebrates, its conversion to clothianidin, through metabolic or environmental processes, can result in greater toxicity. According to Ihara and Matsuda (2018), clothianidin acts as a superagonist in *Drosophila and Xenopus laevis*. These authors suggest that this effect is related to the dynamics of channel opening in nAChRs, observed in single-channel recordings, in which clothianidin tends to induce higher conductance states more frequently than acetylcholine. Thus, even when organisms are predominantly exposed to thiamethoxam, the presence of its metabolite clothianidin can cause significant acute and chronic effects. These findings reinforce that the lower affinity of thiamethoxam for receptors does not, by itself, guarantee a reduction in ecological risk in non-target species (Li et al. 2022).

In addition to receptor-mediated effects, a growing body of evidence indicates that thiamethoxam-induced genotoxicity is largely mediated by oxidative stress (Jameel et al. undefined2020; Elhamalawy et al. 2022; Issa et al. 2025). Exposure to thiamethoxam has been associated with increased production of reactive oxygen species (ROS), lipid peroxidation, and disruption of antioxidant defense systems, including alterations in SOD, CAT, GSH, and GPx activity (Jameel et al. 2020; Elhamalawy et al. 2022; Issa et al. 2025). This redox imbalance is a central mechanism, promoting oxidative DNA damage, as evidenced by increased levels of oxidized nucleotides such as 8-hydroxy-2’-deoxyguanosine (8-OHdG), and contributing to the formation of DNA strand breaks detectable by the Comet assay (Abdel-Razik et al. 2022; Issa et al. 2025). Such damage has been consistently reported across multiple taxa, including fish (Ghaffar undefined 2020), birds (Pan et al. 2021), and mammals (Abdel-Razik et al. 2022; Elhamalawy et al. 2022; Issa et al. 2025), indicating that thiamethoxam can induce systemic genotoxic effects in different tissues.

At the cellular level, thiamethoxam-induced genotoxic stress is also associated with modulation of key signaling pathways involved in cell survival, apoptosis, and inflammation (Wang et al. 2025; Okle et al. 2018). Evidence from vertebrate models indicates that thiamethoxam exposure can alter the expression of genes related to xenobiotic metabolism (e.g., CYP450 enzymes), cell cycle regulation, and stress responses, while also promoting inflammatory signaling (e.g., IL-6) and dysregulating apoptotic pathways (e.g., Bcl-2) (Wang et al. 2025; Pan et al. 2021; Okle et al. 2018). In some cases, high doses have been linked to ferroptosis and mitochondrial dysfunction, suggesting that multiple forms of regulated cell death may be involved (Pan et al. 2021). This expanded mechanistic framework strengthens the biological interpretation of our results and supports the use of sensitive biomarkers to detect early genotoxic events.

The results of the Micronucleus test revealed more pronounced nuclear alterations at the highest thiamethoxam concentration, with a predominance of nuclear buds, anucleated cells, and lobed nuclei. This pattern suggests that the compound’s mutagenicity tends to manifest itself more clearly under conditions of greater cellular stress, that is, at higher concentrations, when the damage exceeds the organism’s repair capacity. Indeed, reports in *Drosophila melanogaster* indicate that thiamethoxam mutagenic effects are more pronounced at the highest concentrations tested (Morais et al. 2017); in *Tradescantia pallida*, the frequency of micronuclei increases as the thiamethoxam dose increases (Morais et al. 2019); and in human cell lines (HepG2 and SH-SY5Y), thiamethoxam exposure results in DNA damage and cytotoxicity that intensify with dose (Şenyildiz et al. 2018). In this sense, the increase in nuclear anomalies may reflect not only chromosomal instability but also the activation of cell death and nuclear restructuring mechanisms associated with the cell cycle. This result broadens the interpretation of the observed effects, indicating that, in addition to direct DNA damage, thiamethoxam also compromises the maintenance of nuclear integrity, which may impact cell viability in later stages of development.

Although the average frequency of micronuclei observed in L. catesbeianus was relatively low (0.92 ± 0.63), this pattern is consistent with findings reported for amphibians and fish exposed to genotoxic agents under acute experimental conditions. Across aquatic vertebrates, micronucleated erythrocytes are generally rare events, even under genotoxic stress, and significant responses are typically detected as fold-increases over low baseline frequencies rather than as high absolute percentages (Arcaute et al. 2014, 2016). The magnitude of the response may vary according to species-specific sensitivity, chemical characteristics, exposure duration, and concentration, but acute exposure assays commonly yield micronucleus frequencies within the same order of magnitude as those observed in the present study (≤ 1%) (Arcaute et al. 2014, 2016). In amphibians, baseline micronucleus frequencies are generally low because micronuclei represent irreversible chromosomal damage that becomes fixed only after cell division, whereas many primary DNA lesions may still be repaired before mitosis (Fenech 2000; Collins 2004). Consequently, short-term exposures often produce more pronounced responses in Comet assay parameters than in Micronucleus frequency, a pattern consistently reported in aquatic vertebrates exposed to pesticides and other mutagens. Comparative studies in fish and clams exposed to direct and indirect mutagens demonstrated clearer and more statistically robust dose–time responses with the Comet assay than with the Micronucleus test, indicating greater sensitivity of the Comet assay for detecting early genotoxic effects (Kim and Hyun 2006). Similarly, acute 24–96 h exposures to organophosphates and carbosulfan in fish produced strong concentration- and time-dependent increases in Comet parameters, whereas Micronucleus responses were comparatively limited (D’Costa et al. undefined 2018). In amphibian tadpoles, exposure to imidacloprid and formulated neonicotinoid products induced pronounced increases in Comet damage indices at concentrations where Micronucleus responses were absent, subtle, or restricted to specific doses and exposure periods, again supporting the higher sensitivity of the Comet assay for detecting transient DNA lesions during short-term exposure (Pérez-Iglesias et al.^2014^; Arcaute et al. 2014).

Nevertheless, even modest but statistically significant increases in micronucleus frequency and other nuclear abnormalities are considered biologically meaningful indicators of clastogenic and aneugenic effects, particularly when associated with oxidative stress, nuclear anomalies, developmental alterations, reduced growth, or behavioral impairment. Reviews and field studies in amphibians emphasize that these biomarkers represent early-warning indicators linking genomic instability to developmental delays, malformations, reduced adaptive potential, and possible population-level impacts (Benvindo-Souza et al. 2020; Silva et al. 2021; Samojeden et al. 2022). This interpretation is supported by the stronger responses observed in the Comet assay. Taken together, these findings highlight the complementary nature of the two biomarkers, since the Comet assay reflects early genotoxic events whereas the micronucleus test indicates the establishment of more persistent chromosomal alterations and mutagenic effects.

In this context, the correlation analyses among the biomarkers (Comet and Micronucleus/other nuclear abnormalities) revealed a positive association between Tail Length, Olive Tail Moment, and the frequency of lobed nuclei, suggesting an interdependence between primary DNA damage and the manifestation of more complex nuclear alterations. The positive correlations observed between Comet assay parameters and the frequency of Micronuclei and other nuclear abnormalities suggest a mechanistic continuum between primary DNA damage and the establishment of irreversible chromosomal alterations. The Comet assay detects early and potentially repairable lesions, such as DNA strand breaks and oxidative damage, representing one of the first cellular responses to genotoxic stress (Collins 2004; Collins et al. 2023). According to Fenech (2000) and Collins (2004), when these lesions are not efficiently repaired before DNA replication or mitosis, they may result in chromosome fragmentation, mitotic spindle dysfunction, or errors in chromosomal segregation, culminating in the formation of micronuclei and abnormal nuclear morphologies detected by the Micronucleus test. Thus, the observed associations indicate that thiamethoxam-induced genomic instability may progress from transient molecular lesions to fixed mutagenic events, reflecting interconnected stages of cellular damage rather than isolated biomarker responses (Ghaffar 2020; Issa et al. 2025). Furthermore, persistent genomic instability may activate cytotoxic pathways such as apoptosis and cell cycle arrest, helping explain the concomitant increase in nuclear abnormalities associated with loss of cellular integrity (El-Din et al. 2023; Issa et al. 2025).

In addition to genetic and chromosomal damage, our results also indicate changes consistent with cytotoxicity (Table 5). This body of evidence is supported by the literature, which indicates that the inability to repair DNA damage can culminate in chromosomal instability or the activation of apoptotic pathways, configuring complementary mechanisms of cellular response to genotoxic stress (Fenech 2000; Norppa 2004). Therefore, our findings reinforce that thiamethoxam compromises not only genomic integrity but also cell viability, suggesting a potential simultaneous mutagenic and cytotoxic effect. These findings broaden our understanding of the cellular effects induced by thiamethoxam, indicating that the interaction between biomarkers and analyses performed should not be interpreted in isolation, but rather as part of a sequence of biological responses to the pesticide.

At the individual and population level, DNA alterations induced by pesticides such as thiamethoxam have the potential to reduce the fitness of anurans, in different biological manifestations such as morphological malformations and behavioral changes, effects that have been demonstrated experimentally in tadpoles exposed to neonicotinoids, including specific reports of decreased size and food impacts (Samojeden et al. 2022). In laboratory and biomonitoring studies, exposure to neonicotinoids has been associated with significant increases in Comet and Micronucleus test parameters in fish and other aquatic vertebrates, as well as in mammals and experimental models, raising concerns about the real effect on exposed vertebrates (Pérez-Iglesias et al. 2014; Şenyildiz et al. 2018; Ayşegül and Turan 2022; Dutta et al. 2024; Mamy et al. 2025). It is also known that factors influencing proliferation, differentiation, or apoptosis can induce embryotoxic or teratogenic effects, and, by extension, may impact later developmental stages, including tadpoles (Samojeden et al. 2022). High frequencies of micronuclei in human lymphocytes have been associated with an increased cancer risk (Bonassi et al. 2007). Similarly, recurrent findings of DNA damage in aquatic organisms using Micronucleus and Comet assays indicate genotoxic and mutagenic exposure, which may be potentially linked to pro-carcinogenic processes, although direct evidence of cancer in these species has not yet been established (Collins 2004; Frenzilli et al. 2009).

Neoplastic transformation involves dysregulated cell proliferation, evasion of apoptosis, cell cycle disruption, neoangiogenesis, and, in advanced stages, invasion and metastasis, classic phenotypes of oncogenesis that are fostered by genomic instability and mutations in regulatory pathways; therefore, pesticide-induced genotoxic events mechanistically increase the risk of tumor transformation (Hanahan and Weinberg 2011). In wild organisms, even subclinical cellular changes can affect body condition, reduce reproductive capacity, and compromise individual survival, with cumulative effects that can affect population dynamics and local viability, a concern especially relevant for anurans due to their aquatic-terrestrial life cycle and skin permeability that favors exposure and internalization of xenobiotics (Mandal et al. 2025; Oliveira et al. 2025).

## Conclusion

Our results demonstrate that thiamethoxam induces relevant acute effects in *L. catesbeianus* tadpoles, including mortality (LC10−96 h = 1070.13 µg a.i./L) and genotoxic (Comet assay - in the range of 31 µg a.i./L), mutagenic, and cytotoxic effects (in the range of 31 and 59 µg a.i./L) - concentrations that can be found in the environment. The Comet assay showed greater sensitivity for detecting primary DNA damage, while the Micronucleus test revealed chromosomal instability and irreversible nuclear alterations.

The genotoxic response observed at 31 µg a.i./L (Comet assay) indicates a non-monotonic pattern consistent with the mechanisms described for neonicotinoids, possibly associated with nAChR and/or the intensification of cytotoxic processes. By integrating lethal responses and cytogenetic biomarkers, this study contributes to filling gaps in knowledge about the impacts of neonicotinoids on anuran amphibians, providing evidence of the susceptibility of *L. catesbeianus* to acute exposure to thiamethoxam, and by extension in aquatic vertebrates. Furthermore, it reinforces the potential of amphibians as sensitive bioindicators of aquatic contamination.

Given the ecological relevance of these findings, future studies should incorporate different species, developmental stages, and biomarkers associated with cholinergic signaling, DNA repair, cell viability, as well as parameters associated with cell death, in order to improve the understanding of non-monotonic responses and strengthen more realistic ecotoxicological risk assessments for neonicotinoids in aquatic ecosystems.

## Supplementary Information

Below is the link to the electronic supplementary material.

Supplementary Material 1

## Data Availability

No datasets were generated or analysed during the current study.
