# Supplementary material for: Environmentally relevant concentrations of thiamethoxam cause cytotoxic and genotoxic damage in bullfrog (Lithobates catesbeianus) tadpoles
Source: Ecotoxicology. 2026 Oct 1;35(8):182. doi: 10.1007/s10646-026-03154-0 (PMC13630812; doi:10.1007/s10646-026-03154-0)
Supplement: Supplementary file 1 [file 10646_2026_3154_MOESM1_ESM.docx]

**Supporting Information**

**Environmentally relevant concentrations of thiamethoxam cause cytotoxic and genotoxic damage in bullfrog (*Lithobates catesbeianus*) tadpoles**

Aline Arantes De Oliveira ^1*^, Miller Caldas Barradas ^2^, Acácio Arlem Tomaz ^3^, Diego da Silva Lima ^4^, João Victor Borges Assis ^5^, Andréa Rodrigues Chaves ^6^, Thiago Bernardi Vieira ^7^, Marta Sofia Monteiro ^8^, Renato Almeida Sarmento ^9^, Althiéris de Souza Saraiva ^10^, Daniela De Melo e Silva ^11^

^1*^ Aline Arantes De Oliveira

Laboratory of Mutagenesis (LABMUT), Institute of Biological Sciences (ICB I), Federal University of Goiás (UFG), and Center of Excellence in One Health (CESU), City: Goiânia, Goiás, Brazil; and research group: Conservation of Agroecosystems and Ecotoxicology (CAE), City: Campos Belos, Goiás, Brazil; and Department of Biology and Center for the Study of the Environment and the Sea (CESAM), University of Aveiro (UA), City: Aveiro, Portugal

+55 62 99987-2461

[alineoliveira@ua.pt](mailto:alineoliveira@ua.pt) / aline_arantes@discente.ufg.br / alinearantesgh@gmail.com

<https://orcid.org/0000-0002-3668-6046>

^2^ Miller Caldas Barradas

Laboratory of Mutagenesis (LABMUT), Institute of Biological Sciences (ICB I), Federal University of Goiás (UFG), and Center of Excellence in One Health (CESU), City: Goiânia, Goiás, Brazil

[millercaldas@discente.ufg.br](mailto:millercaldas@discente.ufg.br)

https://orcid.org/0009-0001-3324-7451

^3^ Acácio Arlem Tomaz

Laboratory of Mutagenesis (LABMUT), Institute of Biological Sciences (ICB I), Federal University of Goiás (UFG), and Center of Excellence in One Health (CESU), City: Goiânia, Goiás, Brazil

acacio_a_tomaz@discente.ufg.br / https://orcid.org/0000-0002-9130-395X

^4^ Diego da Silva Lima

Laboratory of Mutagenesis (LABMUT), Institute of Biological Sciences (ICB I), Federal University of Goiás (UFG), City: Goiânia, Goiás, Brazil

[diego.lima@discente.ufg.br](mailto:diego.lima@discente.ufg.br)

https://orcid.org/0009-0007-5607-9706

^5^ João Victor Borges Assis

Center of Excellence in Molecular Studies, Energy and Petroleum (CEMEP), Institute of Chemistry (IQ), Federal University of Goiás (UFG), City: Goiânia, Goiás, Brazil

joaovictorborgesassis@gmail.com

https://orcid.org/0000-0001-9718-9206

^6^ Andréa Rodrigues Chaves

Center of Excellence in Molecular Studies, Energy and Petroleum (CEMEP), Institute of Chemistry (IQ), Federal University of Goiás (UFG), City: Goiânia, Goiás, Brazil

[andrea_chaves@ufg.br](mailto:andrea_chaves@ufg.br)

https://orcid.org/0000-0002-1600-1660

^7^ Thiago Bernardi Vieira

Laboratory of Chiropteran Studies (LABEQ), Faculty of Biological Sciences (FCB), Federal University of Pará (UFPA), Altamira University Campus, City: Altamira, Pará, Brazil

thiagobernardi007@gmail.com

<https://orcid.org/0000-0003-1762-8294>

^8^ Marta Sofia Monteiro

Department of Biology (DBio) and Center for the Study of the Environment and the Sea (CESAM), University of Aveiro (UA), City: Aveiro, Portugal

mmonteiro@ua.pt

<https://orcid.org/0000-0002-1891-7365>

^9^ Renato Almeida Sarmento

Federal University of Tocantins (UFT), Postgraduate Program in Plant Production (PPGPV), Applied and Functional Ecology Laboratory, National Institute of Science and Technology (INCT), Gurupi University Campus, City: Gurupi – Tocantins, Brazil

rsarmento@mail.uft.edu.br

<https://orcid.org/0000-0002-5379-9595>

^10^ Althiéris de Souza Saraiva

Professor and researcher at the Federal Institute of Goiás (IF Goiano), Campos Belos Campus, research group: Conservation of Agroecosystems and Ecotoxicology (CAE), City: Campos Belos, Goiás, Brazil

[althieris.saraiva@ifgoiano.edu.br](mailto:althieris.saraiva@ifgoiano.edu.br)

<https://orcid.org/0000-0002-8583-9803>

^11^ Daniela De Melo e Silva

Laboratory of Mutagenesis (LABMUT), Institute of Biological Sciences (ICB I), Federal University of Goiás (UFG), and Center of Excellence in One Health (CESU), City: Goiânia, Goiás, Brazil

[danielamelosilva@ufg.br](mailto:danielamelosilva@ufg.br)

<https://orcid.org/0000-0003-0362-0988>

* Correspondence author:

Aline Arantes De Oliveira

Institute of Biological Sciences I (ICB I) - Room 105,

Federal University of Goiás,

City: Goiânia, Goiás, CEP: 74690-900, Brazil

[alineoliveira@ua.pt](mailto:alinearantesgh@gmail.com) / [aline_arantes@discente.ufg.br](mailto:aline_arantes@discente.ufg.br) / alinearantesgh@gmail.com

**Calculations of preparing experimental treatments**

Product information: CRUISER® 350 FS → 350 g tiametoxam / L = 350 mg / mL = 350.000 µg / mL.

Stock solution – nominal concentration (1 L): 100 mg a.i./L (volume of commercial product for 1 L = 0.286 mL).

Experimental design: 7 treatments; 2 L per aquarium; 5 replicates → 10 L per treatment.

| **Table S1** Relationship between nominal concentrations of the lethal concentration test, mass of active ingredient required, and volume of commercial product. | | | |
| --- | --- | --- | --- |
| ***Treatment*** | ***Nominal concentrations (µg a.i./L)*** | ***Mass per 10 L (µg)*** | ***Product volume (mL)*** |
| T1 | 14.4 | 144.0 | 0.00041143 |
| T2 | 36 | 360.0 | 0.00102857 |
| T3 | 90 | 900.0 | 0.00257143 |
| T4 | 225 | 2.250 | 0.00642857 |
| T5 | 562.5 | 5.625 | 0.01607143 |
| T6 | 1406 | 14.060 | 0.04017143 |
| T7 | 3516 | 35.160 | 0.10045714 |
| Total | - | 58.499 | 0.16714 |

| **Table S2** Relationship between nominal concentrations of the subindividual, mass of active ingredient required, and volume of commercial product. | | | |
| --- | --- | --- | --- |
| ***Treatment*** | ***Nominal concentrations (µg a.i./L)*** | ***Mass per 10 L (µg)*** | ***Product volume (mL)*** |
| T1 | 13.4 | 134.0 | 0.0003829 |
| T2 | 26.75 | 267.5 | 0.0007643 |
| T3 | 53.5 | 535.0 | 0.0015286 |
| Total | - | 936.5 µg | 0.0026758 |

Tables S1 and S2 detail, in projection, the calculations of the nominal concentrations and the corresponding volumes of commercial product that would be required for the experiment.

**Added volumes of stock solution (per 2 L aquarium): µL and mL**

- 14.4 µg/L → 288 µL
- 36 µg/L → 720 µL
- 90 µg/L → 1800 µL
- 225 µg/L → 4.500 mL
- 562.5 µg/L → 11.25 mL
- 1406 µg/L → 28.12 mL
- 3516 µg/L → 70.32 mL
- 13.4 µg/L → 268 µL
- 26.75 µg/L → 535 µL
- 53.5 µg/L → 1070 µL

**Materials used**

Automatic pipette P1000 µL

Glass pipette with 25 mL bulb

**Additional analytical information**

See Table 1 in the article

Stock solution (1 L), Sample 4 (A4): 97.3 mg a.i./L.

Intermediate solutions (1 L), Samples 3, 2, 1 (A3, A2, A1): 46 mg/L, 17.0 mg/L, and 4.8 mg/L.

Sub-individual endpoint concentrations: 9.4 µg a.i./L, 31 µg a.i./L and 59 µg a.i./L.

**Chemical analysis (other information)**

Acetonitrile (≥ 99.9%, HPLC gradient grade), formic acid (≥ 98%, ACS reagent grade), and ammonium formate (≥ 97%, analytical grade) were also purchased from Sigma-Aldrich. Methanol (HPLC grade) was supplied by J.T. Baker (Phillipsburg, NJ, USA). Deuterated melatonin (melatonin-d₄, C₁₃D₄H₁₂N₂O₂, 98 atom % D; Sigma-Aldrich) was used as the internal standard (IS). Ultrapure water was produced using a Gehaka Master WFI system (São Paulo, Brazil).

A stock solution of thiamethoxam (1 µg mL⁻¹) was prepared in ultrapure water, and calibration curves were constructed in the range of 5-100 ng mL⁻¹. A stock solution of melatonin-d₄ (1 µg mL⁻¹) was similarly prepared in ultrapure water and added to all standards and samples to achieve a final IS concentration of 50 ng mL⁻¹. Water samples were filtered through 0.22 µm PVDF syringe filters (13 mm) prior to LC-MS/MS analysis. Samples exceeding the calibration range were diluted with ultrapure water to fall within the validated interval.

LC-MS/MS analyses were performed using an Agilent 1290 Infinity II Bio LC system coupled to an Agilent 6495D triple-quadrupole mass spectrometer (Agilent Technologies, Santa Clara, CA, USA). The LC system comprised a high-speed pump (G7132A), thermostatted multisampler (G7137A), and multicolumn thermostat (G7116B). Chromatographic separation was achieved on an InfinityLab Poroshell 120 EC-C18 column (100 × 2.1 mm, 2.7 µm; Agilent) maintained at 40 °C.

The autosampler was kept at 10 °C, with a flow rate of 0.5 mL min⁻¹ and an injection volume of 4 µL. Mobile phase A consisted of water with 0.5% methanol, 0.1% formic acid, and 3.5 mM ammonium formate; mobile phase B consisted of 96% acetonitrile, 2% methanol, and 2% water, with 0.1% formic acid and 3.5 mM ammonium formate. The gradient program for mobile phase B was: 2% B (0-0.2 min), 20% B (1.5-2.5 min), ramp to 100% B (5.0-6.5 min), return to 2% B at 6.51 min, and hold for a total run time of 8.0 min, followed by a 1.0 min post-run for column re-equilibration.

Ionization was carried out using an Agilent Jet Stream electrospray ionization (AJS-ESI) source in positive/negative switching mode. Source parameters were as follows: drying gas flow, 13.0 L min⁻¹; nebulizer pressure, 40.0 psi; sheath gas flow, 11.0 L min⁻¹; drying gas temperature, 250 °C; sheath gas temperature, 350 °C; capillary voltage, 3500 V (positive) and 4000 V (negative); nozzle voltage, 500 V (positive) and 1500 V (negative).

Data acquisition was performed in dynamic multiple reaction monitoring (dMRM) mode. For each analyte, the most intense product ion was used as the quantifier and the second as the qualifier. Scheduled MRM transitions were aligned to retention times with compound-specific detection windows, and dwell times were automatically optimized to ensure adequate data points across chromatographic peaks. Instrument control and data processing were performed using Agilent MassHunter Workstation software (v. 12.1). Optimized LC-MS/MS parameters, including retention times, MRM transitions, and collision energies, are provided in the Table S3.

The LC-MS/MS method was validated as a multiresidue procedure for monitoring pesticide residues. For the animal exposure assays, only thiamethoxam was administered. The absence of other pesticide transitions in water samples confirmed the specificity of thiamethoxam exposure and the robustness of the analytical protocol.

Linearity was evaluated by plotting calibration curves of the peak-area ratio of thiamethoxam to the internal standard over the range of 5-100 ng mL⁻¹. Calibration in ultrapure water yielded a determination coefficient (R²) > 0.995, which was considered acceptable.

The limit of quantification (LOQ) for thiamethoxam was estimated from calibration curves prepared in ultrapure water and calculated as the ratio between the standard deviation (σ) of responses from five blank samples and the slope (S) of the calibration curve (Equation 1).

**Equation 1.**

|  | Equation 1 |
| --- | --- |

Method recovery was assessed by fortifying water samples with thiamethoxam at three concentration levels (5, 25, and 100 ng mL⁻¹). Recoveries were calculated using Equation 2, where *Cₘ* is the measured concentration, *C₀* the native concentration, and *Cₐ* the added concentration.

**Equation 2.**

|  | Equation 2 |
| --- | --- |

| **Table S3** The optimized LC-MS/MS parameters for each analyte | | | | | | |
| --- | --- | --- | --- | --- | --- | --- |
| **Analyte** | **tr ± time window (min)** | **Monoisotopic Mass** | **MRM transitions** | | | |
|  |  |  | **Ionization type of precursor ion** | **precursor ion > product ion (CE, V)** | | |
|  |  |  |  | **Quantifier** | **Qualifier** | **Polarity** |
| 2,4,5-T | 4.88 ± 0.91 | 254 | [M-H]^-^ | 253 > 195 (10 V) | 255 > 197 (10 V) | - |
| 2,4-D | 4.68 ± 0.85 | 220 | [M-H]^-^ | 219 > 161 (15 V) | 221 > 163 (15 V) | - |
| Acephate | 1.77 ± 1.13 | 183 | [M+H]^+^ | 184 > 143 (1 V) | 184 > 49 (1 V) | + |
| Alachlor | 5.36 ± 1.36 | 269 | [M+H]^+^ | 270 > 238 (10 V) | 270 > 162 (20 V) | + |
| Aldicarb | 4.28 ± 1.15 | 190 | [M+NH_4_]^+^ | 208 > 116 (2 V) | 208 > 89 (12 V) | + |
| Ametryn | 4.82 ± 1.17 | 227 | [M+H]^+^ | 228 > 186 (17 V) | 228 > 96 (25 V) | + |
| Atrazine | 4.76 ± 0.96 | 215 | [M+H]^+^ | 216 > 174 (16 V) | 216 > 68 (40 V) | + |
| Benzidine | 1.72 ± 1.15 | 184 | [M+H]^+^ | 185 > 167 (33 V) | 185 > 168 (19 V) | + |
| Carbendazim | 2.47 ± 1.16 | 191 | [M+H]^+^ | 192 > 160 (16 V) | 192 > 132 (32 V) | + |
| Carbofuran | 4.63 ± 1.13 | 221 | [M+H]^+^ | 222 > 123 (30 V) | 222 > 55 (24 V) | + |
| Chlorpyriphos | 5.88 ± 0.99 | 349 | [M+H]^+^ | 350 > 322 (9 V) | 350 > 198 (25 V) | + |
| Cyproconazole | 5.08 ± 1.32 | 291 | [M+H]^+^ | 292 > 89 (60 V) | 292 > 70 (17 V) | + |
| Deisopropylatrazine | 2.69 ± 1.14 | 173 | [M+H]^+^ | 174 > 104 (25 V) | 174 > 68 (33 V) | + |
| Demeton | 4.93 ± 1.14 | 258 | [M+H]^+^ | 259 > 89 (15 V) | 259 > 61 (40 V) | + |
| Desethylatrazine | 3.57 ± 1.31 | 187 | [M+H]^+^ | 188 > 146 (17 V) | 188 > 62 (57 V) | + |
| Desthio-Prothioconazol | 5.17 ± 1.32 | 311 | [M+H]^+^ | 312 > 125 (32 V) | 312 > 70 (28 V) | + |
| Dimethoate | 3.72 ± 1.28 | 229 | [M+H]^+^ | 230 > 199 (5 V) | 230 > 171 (9 V) | + |
| Diuron | 4.80 ± 1.16 | 232 | [M+H]^+^ | 235 > 72 (20 V) | 233 > 160 (24 V) | + |
| Epoxiconazole | 5.18 ± 1.14 | 329 | [M+H]^+^ | 330 > 121 (21 V) | 330 > 101 (53 V) | + |
| Fipronil | 5.40 ± 1.13 | 436 | [M-H]^-^ | 435 > 330 (20 V) | 435 > 278 (25 V) | - |
| Flutriafol | 4.72 ± 1.29 | 301 | [M+H]^+^ | 302 > 123 (33 V) | 302 > 70 (61 V) | + |
| Melatonin_d4* | 3.93 ± 0.90 | 236 | [M+H]^+^ | 237 > 178 (21 V) | 237 > 163 (43 V) | + |
| Methamidophos | 0.59 ± 0.48 | 141 | [M+H]^+^ | 142 > 125 (10 V) | 142 > 94 (10 V) | + |
| Metolachlor | 5.37 ± 1.15 | 283 | [M+H]^+^ | 284 > 176 (24 V) | 284 > 134 (32 V) | + |
| Metribuzin | 4.51 ± 1.16 | 214 | [M+H]^+^ | 215 > 187 (15 V) | 215 > 131 (20 V) | + |
| Molinate | 4.66 ± 1.14 | 187 | [M+H]^+^ | 188 > 126 (25 V) | 188 > 83 (16 V) | + |
| Picloram | 2.46 ± 1.11 | 240 | [M+H]^+^ | 241 > 168 (34 V) | 241 > 141 (54 V) | + |
| Simazine | 4.43 ± 1.15 | 201 | [M+H]^+^ | 202 > 132 (22 V) | 202 > 124 (26 V) | + |
| Tebuconazole | 5.27 ± 1.17 | 307 | [M+H]^+^ | 308 > 125 (47 V) | 308 > 70 (40 V) | + |
| Thiamethoxam | 2.87 ± 1.14 | 291 | [M+H]^+^ | 292 > 211 (11 V) | 292 > 132 (20 V) | + |
| Thiodicarb | 4.60 ± 1.16 | 354 | [M+H]^+^ | 355 > 108 (10 V) | 355 > 88 (10 V) | + |

^*^IS (Internal Standard)

| **Table S4** Water quality parameters of *Lithobates catesbeianus* at hour 0. Control 6 hours (T0), Control 96 hours (C0), and experimental groups of tadpoles exposed to increasing concentrations of thiamethoxam insecticide (µg/L). | | | | | |
| --- | --- | --- | --- | --- | --- |
| ***Mean Values ± Standard Deviation*** | | | | | |
| ***Exposure Time (0)*** | ***T0*** | ***C0*** | ***13.4 µg/L*** | ***26.75 µg/L*** | ***53.5 µg/L*** |
| ***pH*** | *7.43* | *7.44±0.007* | *7.42±0.01* | *7.42±0.01* | *7.43±0.01* |
| ***Dissolved oxygen (%)*** | *66* | *66±0.71* | *64±1.53* | *66±0.58* | *66±0.58* |
| ***Conductivity (µS/cm)*** | *149.00* | *150.00±0* | *150.00±0* | *151.67±1.53* | *150.33±1.15* |
| ***Turbidity*** | *1.90* | *1.95±0.071* | *1.83±0.06* | *1.90±0.10* | *1.87±0.12* |
| ***Temperature (ºC)*** | *23.98* | *24.00±0* | *24.05±0* | *24.04±0.02* | *24.02±0.03* |

| **Table S5** Water quality parameters of *Lithobates catesbeianus* after 24 hours of exposure to insecticide thiamethoxam. Control 6 hours (T0), Control 96 hours (C0), and experimental groups of tadpoles exposed to increasing concentrations of thiamethoxam (µg/L). Indicates significant differences compared to the C0 (*p < 0.05*; *Kruskal-Wallis*, followed by *Dunn's* post-hoc test). | | | | | |
| --- | --- | --- | --- | --- | --- |
| ***Mean Values ± Standard Deviation*** | | | | | |
| ***Exposure Time (24h)*** | ***T0*** | ***C0*** | ***9.4 µg/L*** | ***31 µg/L*** | ***59 µg/L*** |
| ***pH*** | *7.440* | *7.415±0.02* | *7.436±0.01* | *7.440±0.01* | *7.453±0.01* |
| ***Dissolved oxygen (%)*** | *66* | *63±3.54* | *65±1.53* | *65±0.58* | *66±0.00* |
| ***Conductivity (µS/cm)*** | *149.000* | *15000±0.00* | *156.333±6.03* | *152.333±2.52* | *150.666±1.53* |
| ***Turbidity*** | *2.000* | *1.850±0.07* | *5.966±0.32* | *10.100±0.20* | *17.433±0.42** |
| ***Temperature (ºC)*** | *24.070* | *24.135±0.06* | *24.096±0.19* | *24.110±0.04* | *24.010±0.04* |

| **Table S6** Water quality parameters of *Lithobates catesbeianus* after 48 hours of exposure to insecticide thiamethoxam. Control 6 hours (T0), Control 96 hours (C0), and experimental groups of tadpoles exposed to increasing concentrations of thiamethoxam (µg/L). *Indicates significant differences compared to the C0 (*p < 0.05*; *Kruskal-Wallis*, followed by *Dunn's* post-hoc test). | | | | | |
| --- | --- | --- | --- | --- | --- |
| ***Mean Values ± Standard Deviation*** | | | | | |
| ***Exposure Time (48h)*** | ***T0*** | ***C0*** | ***9.4 µg/L*** | ***31 µg/L*** | ***59 µg/L*** |
| ***pH*** | *7.520* | *7.310±0.16* | *7.687±0.42* | *7.887±0.02* | *7.767±0.06* |
| ***Dissolved oxygen (%)*** | *78* | *75±0.00* | *85±1.00** | *82±2.65* | *82±1.73* |
| ***Conductivity (µS/cm)*** | *151.000* | *156.500±2.12* | *157.333±7.64* | *158.000±6.00* | *153.667±2.52* |
| ***Turbidity*** | *2.500* | *3.550±0.64* | *8.400±0.72* | *8.933±1.56* | *13.500±0.69* |
| ***Temperature (ºC)*** | *23.950* | *24.045±0.05* | *24.007±0.20* | *23.953±0.09* | *23.983±0.07* |

| **Table S7** Water quality parameters of *Lithobates catesbeianus* after 72 hours of exposure to insecticide thiamethoxam. Control 6 hours (T0), Control 96 hours (C0), and experimental groups of tadpoles exposed to increasing concentrations of thiamethoxam (µg/L). *Indicates significant differences compared to the C0 (*p < 0.05*; *Kruskal-Wallis*, followed by *Dunn's* post-hoc test). | | | | | |
| --- | --- | --- | --- | --- | --- |
| ***Mean Values ± Standard Deviation*** | | | | | |
| ***Exposure Time (72h)*** | ***T0*** | ***C0*** | ***9.4 µg/L*** | ***31 µg/L*** | ***59 µg/L*** |
| ***pH*** | *7.690* | *7.475±0.04* | *7.887±0.07* | *8.033±0.05* | *8.120±0.03** |
| ***Dissolved oxygen (%)*** | *77* | *71±0.07* | *76±0.58* | *75±0.58* | *82±6.08* |
| ***Conductivity (µS/cm)*** | *159.000* | *162.500±2.12* | *160.333±2.89* | *167.000±6.24* | *162.667±1.53* |
| ***Turbidity*** | *3.700* | *3.750±0.21* | *7.450±0.74* | *9.567±0.76* | *14.633±0.91** |
| ***Temperature (ºC)*** | *23.810* | *24.040±021* | *23.940±0.27* | *24.027±0.04* | *23.923±0.06* |

| **Table S8** Water quality parameters of *Lithobates catesbeianus* after 96 hours of exposure to insecticide thiamethoxam. Control 6 hours (T0), Control 96 hours (C0), and experimental groups of tadpoles exposed to increasing concentrations of thiamethoxam (µg/L). *Indicates significant differences compared to the C0 (*p < 0.05*; *Kruskal-Wallis*, followed by *Dunn's* post-hoc test). | | | | | |
| --- | --- | --- | --- | --- | --- |
| ***Mean Values ± Standard Deviation*** | | | | | |
| ***Exposure Time (96h)*** | ***T0*** | ***C0*** | ***9.4 µg/L*** | ***31 µg/L*** | ***59 µg/L*** |
| ***pH*** | *7.67* | *7.63±0* | *7.68±0.01* | *7.69±0.01** | *7.67±0.02* |
| ***Dissolved oxygen (%)*** | *94* | *90±0.01* | *93±0.01** | *91±0.01* | *91±0.02* |
| ***Conductivity (µS/cm)*** | *177.00* | *158.00±32.53* | *173.33±7.23* | *180.00±14.73* | *173.33±5.77* |
| ***Turbidity*** | *4.60* | *4.15±1.20* | *8.37±2.75* | *10.33±1.25* | *9.60±0.96* |
| ***Temperature (ºC)*** | *23.63* | *23.69±0.25* | *23.64±0.34* | *23.81±0.10* | *23.81±0.16* |

| **Table S9** Ammonia (NH₃) concentration (ppm) in the water of *Lithobates catesbeianus* over the time of exposure to thiamethoxam insecticide, 0 to 96 hours. Control 6 hours (T0), Control 96 hours (C0) and experimental groups of tadpoles exposed to increasing concentrations of TMX (µg/L). | | | | | |
| --- | --- | --- | --- | --- | --- |
| ***Mean Values ± Standard Deviation*** | | | | | |
| ***Exposure Time*** | ***T0*** | ***C0*** | ***9.4 µg/L*** | ***31 µg/L*** | ***59 µg/L*** |
| ***0 hours*** | *0.003* | *0.003±0* | *0.001±0.001* | *0.002±0.001* | *0.001±0.001* |
| ***24 hours*** | *0.006* | *0.008±0.004* | *0.003±0* | *0.009±0.002* | *0.003±0* |
| ***48 hours*** | *0.006* | *0.003±0.001* | *0.008±0.005* | *0.018±0* | *0.007±0.002* |
| ***72 hours*** | *0.009* | *0.004±0.002* | *0.013±0.008* | *0.018±0* | *0.018±0* |
| ***96 hours*** | *0.062* | *0.049±0.018* | *0.053±0.015* | *0.080±0.031* | *0.053±0.015* |

Abbreviation: T0 = Control 6 hours, C0 = Control 96 hours.

| **Table S10.** Analytical performance of the LC–MS/MS method. | |
| --- | --- |
| **Performance Parameter** | **Result** |
| Calibration curve | y = 0.047x + 0.0562 |
| Linear range (ng mL^-1^) | 5 - 100 |
| R² | 0.9989 |
| LOQ (ng mL^-1^) | 0.39 |
| **Fortification (ng mL^-1^)** | **Recovery (%)** |
| 5 | 72 |
| 25 | 90 |
| 100 | 99 |

**Figure S1.** Representative total ion chromatograms (TICs) of LC-MS/MS analysis.
